# Farmers and Citizens

**Published:** 1882-01

**Authors:** 


**FARMERS AND CITIZENS.**—An extended series of observations seems to have warranted two conclusions, both adverse to commonly received opinions:

First. There are more persons in lunatic asylums from the country than from the town.

Second. The average of human life is greater in the largest cities, than in the country adjoining; yet farmers eat plain, fresh food, take abundant exercise, retire early, and get up by daylight, breathing the pure out-door air for at least half their existence. On the other hand, citizens retire late, rise late, eat food and fruits one, two, or a dozen days' old; are indoors three fourths if not nine tenths of their time, breathing an air vitiated by furnace-heat and a variety of other causes, and take comparatively little exercise.

It is practically useful to note some of the general reasons which may very rationally be considered as explanatory of such results.

The universal tendency of concentration of thought upon one subject is to monomania, madness; this is so palpable a fact that argument is not necessary. When, therefore, the subjects of thought are few in number, this same tendency exists. The weather, the crops, the market, is the idol trinity of most farmers; in a wide sense, they think, talk, dream about nothing else with any special interest; all besides is secondary, and if by any novelty the mind is compelled out of its wonted track, it soon relapses into the old tread-mill circle, into the same rut of ages gone. In great cities this destructive concentration is almost an impossibility; the morning papers, the prices current, the stock-markets, the accidents, the wars of nations, the exhibitions of curious and stirring things, keep the mind on the look-out; in fact, almost too active; there is scarcely enough time for needed rest. The day begins with running over the state of the world, as exhibited in the newspaper. From nine until four the whole mind is absorbed in matters of business; from that until near midnight, there is a comparative abandon to dinner, to social ties, to giving or receiving visits from acquaintances, friends, and kindred, in going to the concert, the lecture, the opera, to evening parties, or other sources of agreeable diversions or profitable intercommunions.

The farmer, glorying in his health and strength, thinks his constitution impregnable; scouts at method and system and precaution, considering them as nothing but doctors' whims and old women's notions. He believes in eating hearty suppers and late; he has done it all his life, and is not dead yet, and resolves so to continue until the end of the chapter, when some morning the news goes round, "Died last night" of apoplexy, cholera-morbus, cramp colic, or the like. At other times bilious fever carries him from health to the grave in ten days, in consequence of going to sleep in the entry or on the front-stoop after a hard day's work; or he brings on some other malady by damp feet, bad cookery, neglecting the calls of nature, or deliberately postponing them. The citizen, on the contrary, has more or less informed himself on these matters, both by reading and observation; he is compelled to pay deference to nature's laws; he knows that their infraction is attended with certain penalties, and his better judgment leads him to estimate properly the value of a wise course of life; while all the time he is relieved from the necessity of encountering great exposure to heat and cold, of excessive and exhausting physical efforts, which accidents and the hurry of the seasons impose on those who cultivate the soil.

Farmers will become healthier in body and in mind, in proportion as agricultural papers are taken, for several reasons: these publications uniformly contain a large amount of unexceptionable family reading, as to health, temperance, and sound morals; they will also gradually waken up the mind of farming people to experiments, to what is often sneeringly styled "scientific farming." Every day the helter-skelter mode of agriculture is becoming less and less remunerative; every day it is becoming more and more necessary to study the laws of vegetable growth, the habitudes and needs of plants and grains and trees; and in proportion as this is done, and the analysis of soils becomes an indispensable pre-requisite, there will be a world of novelty and light to break in upon the farming mind to interest, electrify, and enrich. The time will come when to attempt the successful management of a farm, large or small, without some considerable practical knowledge of chemistry and botany and geology, will be considered the extreme of Quixotism. Meanwhile, let farmers and farmers' wives, with their children, bear in mind that to diminish the chances of a dyspeptic or bilious madness, or a premature death from acute disease, they should practice habits of personal cleanliness and bodily regularity; should eat only at regular hours, not oftener than thrice a day, and never between meals, swallowing not an atom after sundown; eat always slowly and with great deliberation; take nothing for the last meal of the day beyond some cold bread and butter and a single cup of water or warm drink, so as to throw the main meal to breakfast or dinner, thus having all the exercise of the day to "grind it up," to convert it into healthful nutriment. Avoid damp clothing and cold or wet feet; keep out of even the slightest draught of air after all forms of exercise; and all the while practice, as to the body, regularity, temperance, and self-denial; while, as to the mind, cultivate a cheerful spirit, a courteous temper, and a loving heart. The great general idea is this, that as between farmers and citizens of the largest cities, the chances are in favor of the latter as to length of life and mental integrity; that less bodily exercise and more mental activity bring better results in the long run than more exercise and less mental activities; that what tends to waken up and divert the attention, is quite as indispensable to our well-being as bodily activities; that Barnumizing (keeping us waked up to new things and strange) is an institution for health, as well as the gymnasium and the Central Park.

